# Gut Microbiota Is Involved in Alcohol-Induced Osteoporosis in Young and Old Rats Through Immune Regulation

**DOI:** 10.3389/fcimb.2021.636231

**Published:** 2021-07-14

**Authors:** Ming Cheng, Bo Tan, Xiaojing Wu, Feng Liao, Fei Wang, Zuoyao Huang

**Affiliations:** ^1^ Department of Rehabilitation, Jinniu District People’s Hospital of Chengdu, Chengdu, China; ^2^ Department of Orthopedics, Sichuan Academy of Medical Sciences & Sichuan Provincial People’s Hospital, Chengdu, China; ^3^ Department of Orthopaedics, Jinniu District People’s Hospital of Chengdu, Chengdu, China

**Keywords:** alcohol-induced osteoporosis, gut microbiota, RANKL, bone resorption, immunoreaction, age-related

## Abstract

Long-term and excessive alcohol consumption are risk factors for osteoporosis. Excessive drinking can reduce bone density and also cause imbalance of gut microbiota. And gut microbiota can affect bone metabolism through various mechanisms, and the regulation of gut microbiota is closely related to age. However, the effects of gut microbiota on alcohol-induced osteoporosis at different ages are unclear. In this study, young and old rats were used to induce osteoporosis by long-term alcohol consumption, and alcohol metabolism, bone morphology, bone absorption and immune activity of rats were analyzed to determine the effects of alcohol on rats of different ages. In addition, changes of gut microbiota in rats were analyzed to explore the role of gut microbiota in alcohol-induced osteoporosis in rats of different ages. The results showed the ability of alcohol metabolism was only associated with age, but not with alcohol consumption. Long-term alcohol consumption resulted in the changes of bone metabolism regulating hormones, bone loss, activation of receptor activator of NF-*κ*B ligand (RANKL) signaling and inflammatory response. And osteoporosis was more severe in old rats than young rats, suggesting that alcohol-induced osteoporosis is age-related. In addition, long-term drinking also affected the composition of gut microbiota in rats, with a significant increase in the proportion of pro-inflammatory microorganisms. Overall, this study found that long-term alcohol consumption induced osteoporosis and affected the composition of gut microbiota. And alcohol can activate T lymphocytes directly or indirectly by regulating the changes of gut microbiota to produce cytokines, and further activate osteoclasts. In addition, the osteoporosis was more severe in the old rats than young rats, which may be due to the higher diversity and stronger regulation ability of gut microbiota in young rats compared with old rats.

## Introduction

Osteoporosis (OP) is a skeletal disorder characterized by reduced bone mass, altered bone microstructure, increased bone fragility and fracture risk (Lamichhane, 2005). Long-term and excessive alcohol consumption are risk factors for OP (Sotorník, 2016). Evidence has shown that alcohol has multiple direct toxic effects on bone cells (Wang et al., 2003), which can change the activity and proliferation of osteoblasts in a dose-dependent manner (Friday and Howard, 1991). Alcohol also directly or indirectly alters bone mineral metabolism, including parathyroid hormone, vitamin D, testosterone, and cortisol levels (Gaddini et al., 2016). The incidence of osteonecrosis, osteoporosis, and fractures caused by alcohol abuse and alcohol dependence is gradually increasing (Maurel et al., 2012). Alcohol-induced osteoporosis (AOP) is a disorder of systemic bone metabolism, which belongs to secondary OP (Abukhadir et al., 2013) and is very common in clinical practice. In addition, with the increase of age, the rate of bone turnover increases at the tissue level, leading to damaged osteoblast bone formation and increased osteoclast bone resorption, thus significantly increasing the incidence of osteoporosis (Khosla and Riggs, 2005; Manolagas and Parfitt, 2010).

Gut microbiota affects endocrine cells, the enteric nervous system and the immune system, and also can participate in the regulation of bone metabolism (Ohlsson and Sjögren, 2015). The gut microbiota becomes more diverse and variable with advancing age, and age-related gut microbiota dysregulation affects the health and longevity of the host (Kim and Jazwinski, 2018). The imbalance of gut microbiota can change the alkalinity of the intestinal tract and affect the absorption of calcium and vitamin D (Locantore et al., 2020). And gut microbiota may change the immune state of bone, thus affecting the generation of osteoclasts, but its immune regulation ability of the body will gradually weaken as the age increases (Ticinesi et al., 2019). Moreover, it has been found that alcohol can cause changes to the gut microbiota composition and is closely related to personal health (Wang et al., 2020). However, the relationship between AOP and gut microbiota in different ages was not clear. We speculated that the metabolic process of alcohol affected the composition of gut microbiota, and the change of gut microbiota activated T lymphocytes in bone marrow to produce cytokines, thus affecting the differentiation of osteoclasts, moreover this effect may be closely related to age.

To test this hypothesis, the young and old rats were selected for the study as a comparison, and the alcohol metabolism ability, bone morphology, bone resorption, immune activity of different age rats were compared after long-term drinking, to make clear the difference of alcohol influence at different ages. Furthermore, the changes of gut microbiota were analyzed to explore the role of gut microbiota in the occurrence of AOP at different ages. It is of great significance to analyze the potential regulating effects of gut microbiota in the occurrence of age-related AOP, which can provide a new breakthrough point for AOP.

## Materials and Methods

### Animals Experiments

The male 8-week-old SD rats (young rats) and 72-week-old SD rats (old rats) with specific-pathogen-free (SPF) grade were obtained from Institute of Laboratory Animals of Sichuan Academy of Medical Sciences & Sichuan Provincial People’s Hospital (Chengdu, China), and housed under standard laboratory animal conditions. The experiment and all disposals were in accordance with the guidelines of Sichuan Province Experimental Animal Management Committee and National Institutes of Health Guide for the Care.

### Experimental Design

Twelve young or old rats were individually divided into control and alcohol group. The alcohol group rats were fed a Bio-Serv Liquid Rat Diet LD82 (Bio-Serv, Frenchtown, NJ) containing 5.0% (weight/volume) alcohol (35% alcohol-derived calories) for 12 weeks and an adaptive diet for one-week before the start of experiments which the dietary concentration of ethanol was increased from 0% to 35%. Chronic intake of this diet was shown to induce pathologies commonly seen in chronic severe alcohol consumers (Okazaki et al., 2013). The control rats were fed the isocaloric liquid diet with maltose-dextran substituted for ethanol. The rats in the alcohol group were allowed unrestricted access to the alcohol-containing liquid diet. The intake of the pair-fed control rats was strictly limited to the amount ingested on the previous day by the pair-matched ethanol-fed rats to ensure that the calorie intakes of the two groups were the same. After 12 weeks pair feeding, rats were rendered unconscious by CO_2_ inhalation and sacrificed. Blood and femurs of the rats were collected for further analysis. The right femurs were removed from the adjacent tissues and frozen at -80°C for molecular and other detections, and the left femurs were preserved with 4% paraformaldehyde for histological study.

## Biochemical Analysis

Serum was isolated by centrifugation (2000 g, 10 min) with 3K30 Laboratory Centrifuge (Sigma, Bremen, Germany). Supernatant was collected and stored at -20°C for further analysis of the indicators of alcohol metabolism, bone resorption formation and inflammatory cytokines in rat serum. The levels of alcohol dehydrogenase (ADH), acetaldehyde dehydrogenase (ALDH), alkaline phosphatase (ALP), calcium (Ca) and phosphorus (P) were detected by colorimetric method with kit purchased from Nanjing Jiancheng Bioengineering Institute. In addition, the levels of tartrate-resistant acid phosphatase 5b (TRACP-5b), osteocalcin (OCN), calcitonin (CT), osteoprotegerin (OPG), testosterone (T), estradiol (E2), lipopolysaccharides (LPS), tumor necrosis factor-*α* (TNF-*α*), interferon-*γ* (IFN-*γ*) and interleukin 17A (IL-17a) were detected by ELISA with a double antibody sandwich enzyme-linked immunosorbent method (MultiSciences, China). All the operations were carried out in strict accordance with the manufacturer’s protocols, and each sample was repeated three times.

### Histopathological Examination

The left femurs were fixed in 4% paraformaldehyde, and decalcified in 15% EDTA decalcifying liquid for 48 h. The femurs tissues were then embedded in paraffin wax and cut into 5 *μ*m sections. The sections were stained with hematoxylin-eosin (H&E) staining and TRAP staining for pathological studies. The areas of trabecular bone were quantified by Image J analysis software version 1.8.0 (National Institutes of Health) and the numbers of osteoclasts and osteoblasts were counted.

## Immunohistochemical Staining

The tissue sections were dewaxed and quenched endogenous peroxidase activity by 3% hydrogen peroxide. Then the anti-receptor activator of NF-*κ*B ligand (anti-RANKL) antibody (ab216484, 1:500) was incubated with tissue sections overnight at 4°C and the biotinylated secondary antibody (1:1000) at 37°C for 30 min. Diaminobenzidine was used for histochemical reactions and hematoxylin re-staining. Histological examination was performed on a light microscope (Nikon, Tokyo, Japan) and the relative expression of RANKL was quantified.

## Western Blot Analysis

The protein expressions of p-glycoprotein 65 (p65), phosphorylated p65 (p-p65), RANKL, macrophage colony-stimulating factor (M-CSF), nuclear factor of activated T-cells cytoplasmic 1 (NFATc1) and c-Fos in femur tissues were detected by western blot analysis. Proteins were extracted from tissue homogenize with radioimmunoprecipitation assay buffer (RIPA) with 1 mM phenylmethanesulfonyl fluoride (PMSF) (Solarbio, China). Then the protein concentration was quantified by the BCA protein assay kit (Solarbio, China) and SDS-PAGE electrophoresis was performed. And the anti-p65 antibody (sc-8008), anti-p-p65 antibody (sc-166748), anti-M-CSF antibody (sc-365779), anti-NFATc1 antibody (sc-7294) and anti-c-Fos antibody (sc-166940) from Santa Cruz Biotechnology (USA) were used for immune reaction. Finally, the proteins were visualized on Tanon-5200 Chemiluminescent Imaging System (Tanon, China), and the relative protein expression of target protein to *β*-actin was calculated.

## Flow Cytometry

The femur single-cell suspension was prepared by enzyme treatment method for T lymphocyte typing, and the dosage of rats flow cytometric specific antibody (univ-bio, China) was calculated according to the single cell count results. Antibodies were added into a 15 mL centrifuge tube with the single cell suspension in a total reaction system of 1 mL. Antibodies were fully mixed and incubated in the dark at 4°C for 30 min. CD4/CD3, CD4/IL-17 and CD4/CD25/Foxp3 were stained separately. After staining, 10 mL PBS was added for washing, and the supernatant was discarded after centrifugation at 1000 rpm at room temperature for 5 min. The cells were resuspended with 1 mL staining buffer and filtered in a 40 µm sterile screen. The proportion of CD3^+^ CD4^+^ T cells, IL-17^+^ CD4^+^ T cells (Th17) and Foxp3^+^ CD4^+^ T cells (Treg) were analyzed by flow cytometry (Becton, Dickinson and Company, USA).

## DNA Isolation and 16S rRNA Sequencing

Intestinal contents of the rats were collected when rats were sacrificed, fecal DNA was extracted, 16S rRNA sequencing and bacterial quantitative polymerase chain reaction (qPCR) was conducted to analyze the difference and diversity of fecal flora.

## Statistical Analysis

Data were analyzed using SPSS 19.0 (IBM, USA) statistical analysis software, and were shown as mean ± SD. The Student’s T-test was used to compare between two groups. *P* < 0.05 was considered significant.

## Results

### Alcohol Metabolism in Young and Old Rats

Male SD rats at eight and seventy-two weeks were paired with 5% ethanol or maltose-control liquid diets for 12 weeks. The results showed that the levels of ADH and ALDH in the old rats were significantly lower than that of the young rats both in the control and alcohol group (*P* < 0.05), suggesting that the ethanol metabolism ability of the old rats was weaker than that of the young rats. However, the alcohol intake had no significant effects on the enzyme activity of ethanol metabolism in the old or young rats (**Table 1**).

**Table 1:** Alcohol metabolism in young and old rats.

Parameters	Young rats		Old rats	
	Control	Alcohol	Control	Alcohol
ADH (U/mL)	26.310 ± 0.454	25.709 ± 0.649	24.403 ± 0.432^#^	23.519 ± 0.573^#^
ALDH (U/mL)	16.296 ± 0.624	15.743 ± 0.129	15.163 ± 0.272^#^	14.695 ± 0.649 ^#^

Data were shown as mean ± SD. ^#^P < 0.05, the same group compared with young rats. ADH, alcohol dehydrogenase; ALDH, acetaldehyde dehydrogenase.

### Bone Morphology in Young and Old Rats

**Figure 1:**
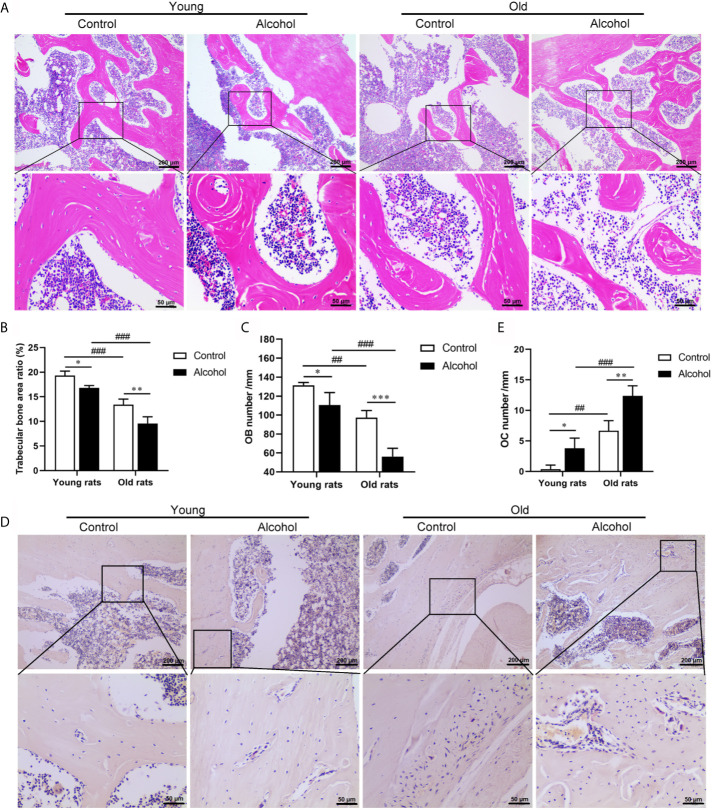
Effects of alcohol on bone morphology in young and old rats. **(A)** Bone tissue was observed by HE staining, Magnification, x100 and x400; **(B)** Trabecular bone area ratio; **(C)** Osteoblasts numbers; **(D)** Osteoclasts was observed by TRAP staining, Magnification, x100 and x400; **(E)** Osteoclasts numbers. Data were shown as mean ± SD. n = 6, ^*^ *P* < 0.05, ^**^ *P* < 0.01, ^***^ *P* < 0.001, compared with control of the same age, ^#^ *P* < 0.05, ^##^ *P* < 0.01, ^###^ *P* < 0.001, the same group compared with young rats.

To determine the effects of alcohol on bone morphology in rats of different ages, histopathological examinations were performed on bone tissue of rats. The results were found after 12 weeks of long-term drinking, compared with control rats, there were a decrease in the micro cracks of the trabeculae bone (**Figure 1A**) and a remarkable decrease in the area ration of trabecular bone (**Figure 1B**) and osteoblasts (**Figure 1C**) in AOP rats (*P* < 0.05). In addition, there were a large number of osteoclasts on the surface of bone trabeculae in AOP rats, which represent obvious bone resorption (**Figures 1D, E**, *P* < 0.05). However, the reduction in trabecular bone area and osteoblasts and the increase of osteoclasts in older rats, both in the control group and the alcohol group, was more pronounced than young rats (*P* < 0.05), suggesting that bone loss after long-term drinking was related to age.

### Osteopenia-Related Serum Biochemical Parameters in Young and Old Rats

To further verify the effects of alcohol on bone resorption and bone formation in rats of different ages, serum biochemical parameters were investigated by ELISA (**Table 2**). And the present results revealed that alcohol-induced osteopenia led a rise in serum tartrate-resistant acid phosphatase 5b (TRAP-5b) level, as compared to the control group both in young and old rats. However, the serum levels of calcium (Ca), phosphorus (P), and other bone turnover markers including alkaline phosphatase (ALP) and osteocalcin (OCN) had no change in young or old rats after long-term drinking. Interestingly, alcohol was found to have different effects on serum levels of osteoprotegerin (OPG), testosterone (T) and estradiol (E2) in rats of different ages. In young rats, the serum levels of OPG and E2 were significantly increased and the level of T was not significantly changed, while OPG and E2 in old rats showed no significant changes and T level was decreased after alcohol treatment. Moreover, age-related differences in some biochemical parameters were found in this study. The old rats in control or alcohol group exhibited a significant decline in serum levels of Ca and OCN compared with young rats treated with the same way. Initially, the older control rats had a lower calcitonin (CT) level and a higher OPG level than young control rats, but there was no difference in CT and OPG between young and old rats after long-term drinking. Moreover, lower serum level of T was found in old alcohol rats compared with young alcohol rats.

**Table 2:** Osteopenia-related serum biochemical parameters in young and old rats after long-term drinking.

Parameters	Young rats		Old rats	
	Control	Alcohol	Control	Alcohol
Ca (mmol/L)	1.163 ± 0.033	1.094 ± 0.035	1.048 ± 0.064^#^	0.988 ± 0.063^#^
P (mmol/L)	2.777 ± 0.177	2.768 ± 0.077	2.790 ± 0.095	2.723 ± 0.183
ALP (U/100mL)	19.616 ± 0.959	19.782 ± 0.980	19.218 ± 0.588	19.719 ± 0.942
TRACP-5b (U/L)	28.520 ± 0.755	30.413 ± 0.847^*^	29.683 ± 0.451	31.397 ± 0.729^*#^
OCN (ng/mL)	1.513 ± 0.035	1.483 ± 0.015	1.440 ± 0.036^#^	1.393 ± 0.031^#^
CT (pg/mL)	125.547 ± 2.437	123.660 ± 1.206	121.423 ± 2.223^#^	120.393 ± 0.588
OPG (pg/mL)	821.100 ± 17.582	885.753 ± 9.057^*^	856.863 ± 27.108^#^	871.970 ± 14.575
T (ng/mL)	2.283 ± 0.012	2.213 ± 0.049	2.187 ± 0.076	2.083 ± 0.061^*#^
E2 (pmol/L)	8.793 ± 0.393	9.630 ± 0.350^*^	9.083 ± 0.356	9.333 ± 0.103

Data were shown as mean ± SD. ^*^P < 0.05, compared with control of the same age, ^#^P < 0.05, the same group compared with young rats. Ca, calcium; P, phosphorus; ALP, alkaline phosphatase; TRAP-5b, tartrate-resistant acid phosphatase 5b; OCN, osteocalcin; CT, calcitonin; OPG, osteoprotegerin; T, testosterone; E2, estradiol.

### Osteoclast Activation Signaling Pathway in Young and Old Rats

**Figure 2:**
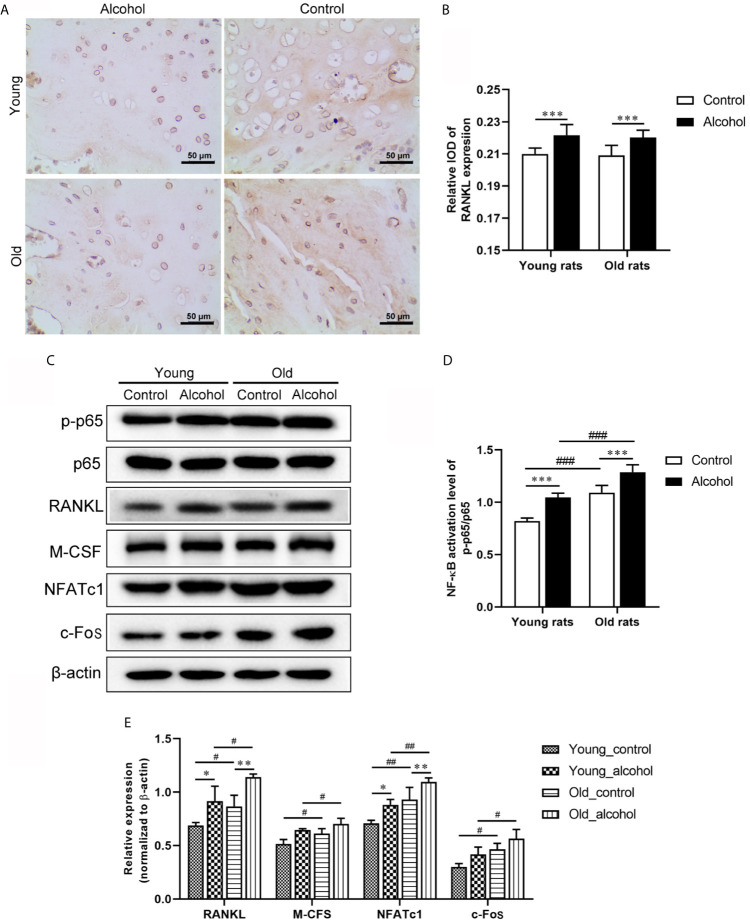
Effects of alcohol on osteoclast activation signaling pathway in young and old rats. **(A)** The expression of RANKL in trabecular bone was observed by IHC; **(B)** The relative IOD of RANKL expression in IHC; **(C)** The protein expression of osteoclast activation signaling pathway was detected by WB; **(D, E)** Densitometry analysis of the intensity of the protein bands. Data were shown as mean ± SD. n=6, ^*^ *P* < 0.05, ^**^ *P* < 0.01, ^***^ *P* < 0.001, compared with control of the same age; ^#^ *P* < 0.05, ^##^ *P* < 0.01, ^###^ *P* < 0.001, the same group compared with young rats.

The expression of RANKL in rats was detected by IHC and WB, and both IHC and WB results showed that the expression of RANKL was significantly increased in young and old rats after long-term drinking (*P* < 0.05). In addition, the protein expressions of p-p65/p65, M-CFS, NFATc1 and c-Fos detected by WB were significantly increased after long-term drinking and the protein expressions were even higher in old rats than in young rats (**Figure 2**), indicating that alcohol intake promoted RANKL signaling activation, and the activation of RANKL promoted by alcohol was age-dependent.

### Immunoactivity in Young and Old Rats

**Figure 3:**
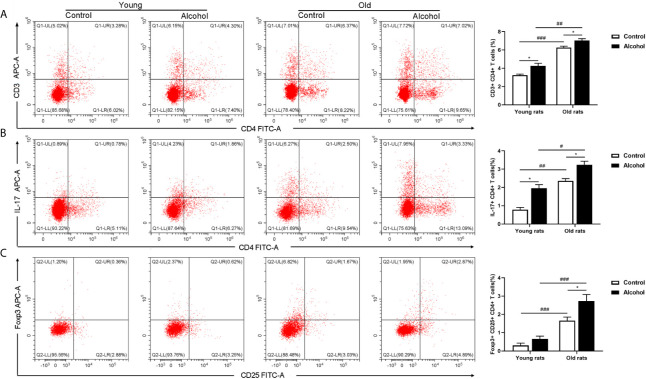
Effects of alcohol on activation of CD4 T lymphocyte in bone marrow of young and old rats. **(A)** The proportion of CD3^+^ CD4^+^ T cells in bone marrow was detected by flow cytometry; **(B)** The proportion of Th17 in bone marrow; **(C)** The proportion of Treg in bone marrow. Data were shown as mean ± SD. n = 6, ^*^ *P* < 0.05, compared with control of the same age; ^#^ *P* < 0.05, ^##^ *P* < 0.01, ^###^ *P* < 0.001, the same group compared with young rats.

**Figure 4:**
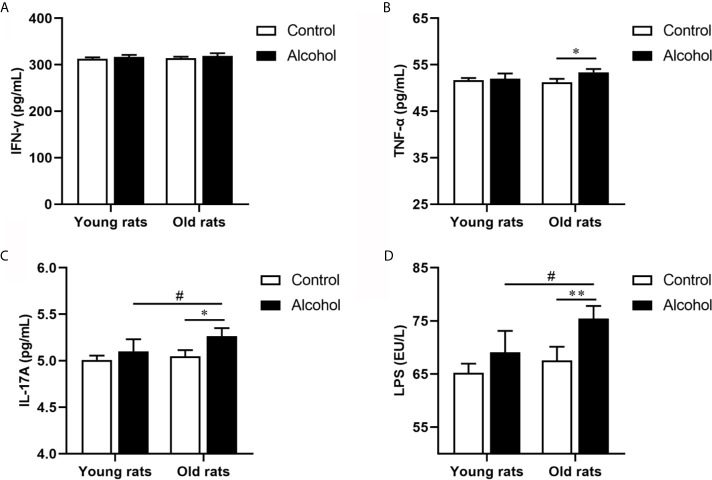
Effects of alcohol on serum inflammatory cytokine in young and old rats. **(A)** The expression of IFN-*γ* in serum; **(B)** The expression of TNF-*α* in serum; **(C)** The expression of IL-17A in serum; **(D)** The expression of LPS in serum. Data were shown as mean ± SD. n = 6, ^*^ *P* < 0.05, ^**^ *P* < 0.01, compared with control of the same age; ^#^ *P* < 0.05, the same group compared with young rats.

The proportion of CD4 T lymphocytes in bone marrow of rats was detected by flow cytometry, and results found that the proportion of CD3^+^ CD4^+^ and Th17 both in young and old rats were significantly increased after long-term drinking, while alcohol only significantly increased the proportion of Treg in the old rats, and the proportion of CD3^+^ CD4^+^ T cells, Th17 and Treg in old rats were significantly higher than that in young rats (*P* < 0.05, **Figure 3**), indicating that the activation of T lymphocytes in bone marrow was not only affected by alcohol intake, but also affected by age. In addition, the serum inflammation cytokines in rats were detected by ELISA, it was found that long-term drinking had no significant effects on the expression of IFN-*γ*、TNF-*α*、IL-17A and LPS in the serum of young rats, while the expressions of TNF-*α*、IL-17A and LPS in the serum of old rats were significantly increased by alcohol (*P* < 0.05). Moreover, the expressions of IL-17A and LPS were significantly higher in the old rats than those in the young rats (*P* < 0.05, **Figure 4**). The results showed that long-term drinking could promote the occurrence of inflammatory response in old rats.

### Gut Microbiota of Young and Old Rats

**Figure 5:**
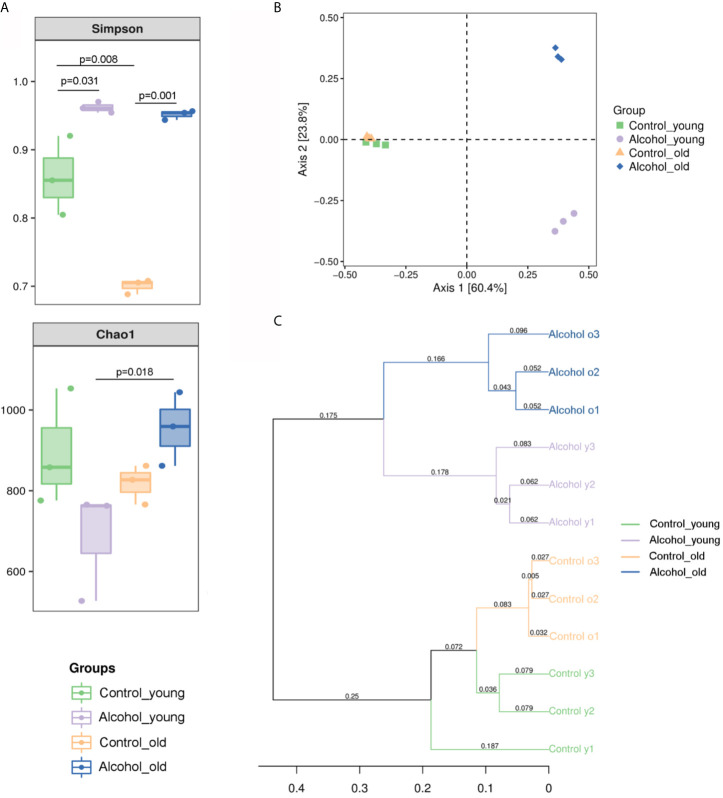
Richness and diversity of fecal microbiota. **(A)** The *α* diversity of fecal microbiota in rats; **(B)** PCoA analysis of Bray-Curtis distances within PC1 and PC2 axes in rats; **(C)** UPGMA cluster analysis based on Bray-Curtis distance in rats. n = 3.

The 16S sequencing-based analysis of gDNA extracted from feces was performed to determine the effects of long-term drinking on gut microbiota of young and old rats. Results showed that the Simpson index of *α* diversity in young rats was significantly higher than that of the old control group, indicating that there were differences in fecal flora diversity between young and old rats. And the Simpson index increased significantly both in young and old rats after long-term alcohol intake, indicating that alcohol intake promoted the increase of fecal flora diversity in rats. In terms of Chao1 index, there was no significant difference between young and old rats, and the results showed that alcohol seemed to increase the Chao1 index in the old rats and decrease it in the young rats, but there was no significant difference in this change. Only differences in Chao index were found between the old alcohol group and the young alcohol group, but it also indicating that alcohol was more likely to promote the increase of microflora richness in old rats (**Figure 5A**). According to Bray-Curtis PCoA analysis as shown in **Figure 5B**, there was no significant difference in the spatial distribution of gut microbiota between young and old control rats, but alcohol intake would promote the change of fecal bacterial flora between young and old rats in different spatial directions, indicating that the changes of bacterial flora composition were different between the young and old rats after long-term alcohol intake. In addition, based on the UPGMA cluster analysis at the Bray-Curtis distance, the composition and structure of the bacterial flora of young and old rats before and after drinking were significantly different in classification and aggregation (**Figure 5C**). All these results suggested that *α* diversity of fecal bacterial flora in young and old rats increased significant after alcohol intake, but *β* diversity of fecal bacterial flora in old and young rats was different, suggesting that alcohol intake had different effects on bacterial composition in young and old rats.

**Figure 6:**
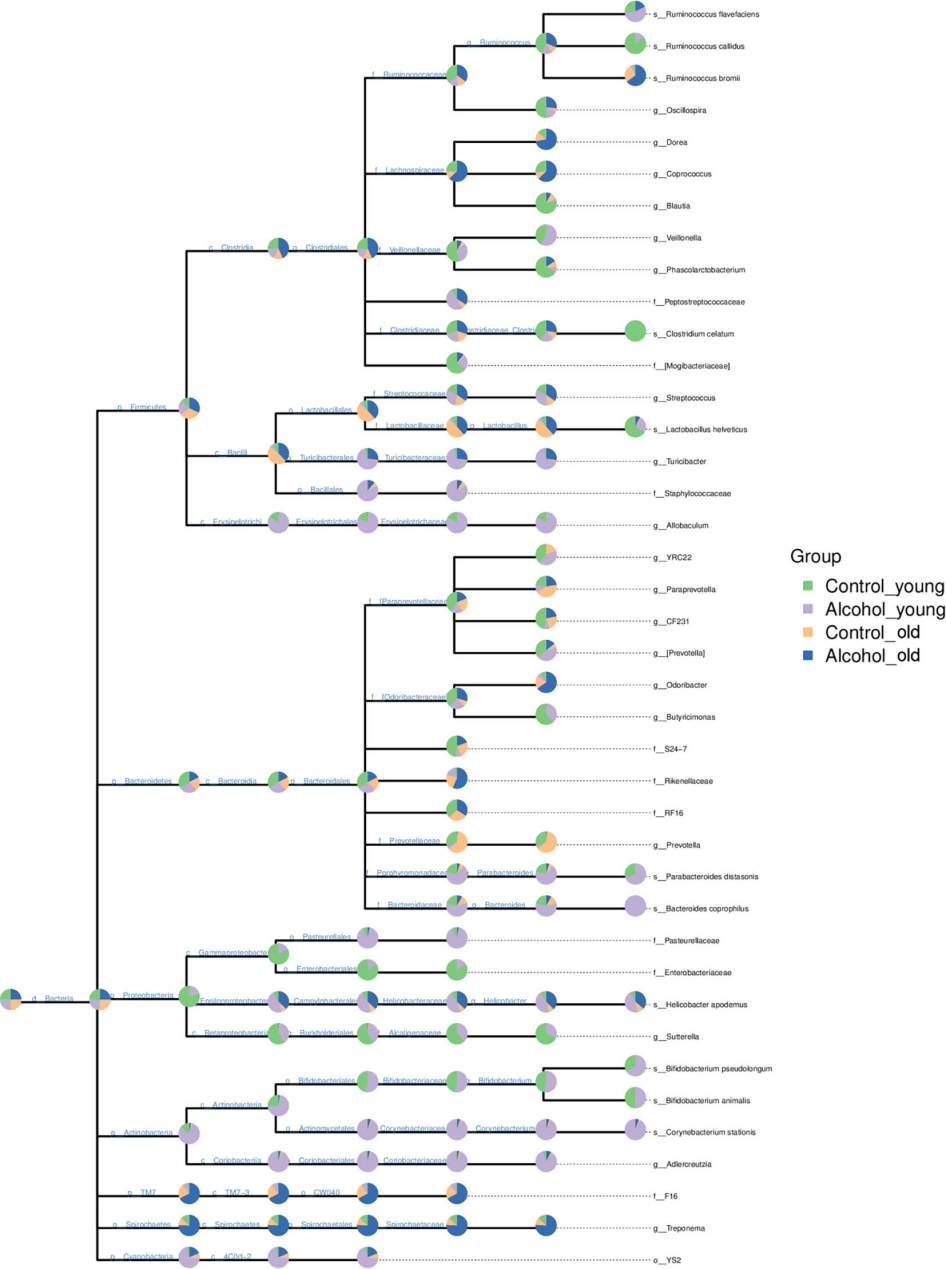
Tree species classification of young and old rats. The pie chart of different colors represents different groups in that classification unit. The size of the radius denotes the tag number accounts for the proportion of the total tag; the larger the radius, the higher the abundance. n = 3.

**Figure 7:**
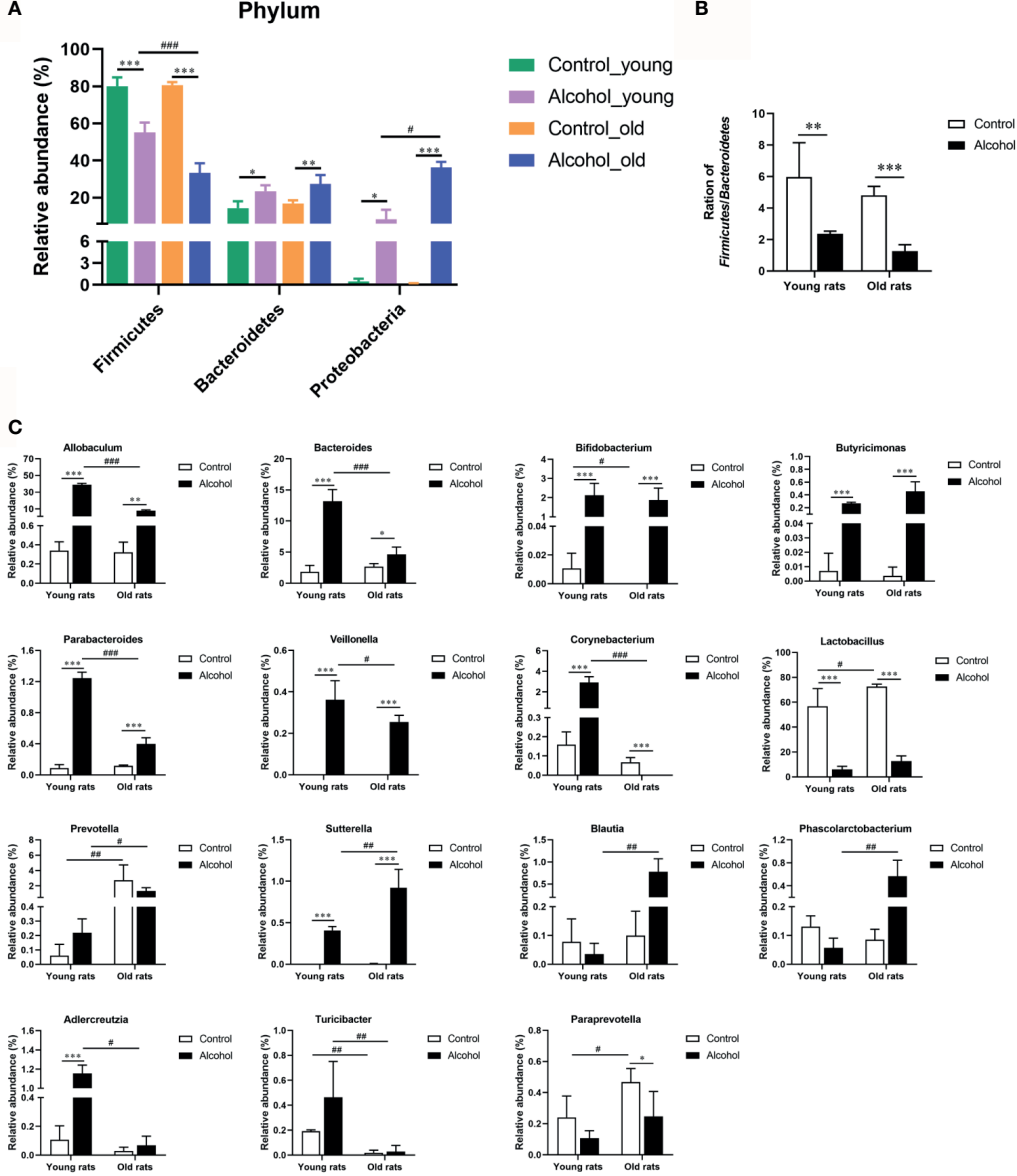
Effects of alcohol on the microflora in fecal stool samples of young and old rats. **(A)** The relative abundance of each phylum; **(B)** The ration of Firmicutes/Bacteroidetes; **(C)** The relative abundance at the genus level. Data were shown as mean ± SD. n=3, ^*^ *P* < 0.05, ^**^ *P* < 0.01, ^***^ *P* < 0.001, compared with control of the same age; ^#^ *P* < 0.05, ^##^ *P* < 0.01, ^###^ *P* < 0.001, the same group compared with young rats.

In order to further analyze the effects of alcohol on the gut microbiota, the difference of taxonomic abundance between different groups was performed by tree species classification, as shown in **Figure 6**, and the analysis of relative abundance was shown in **Figure 7**. Some bacteria in the fecal samples, such as Firmicutes, Bacteroidetes and Proteobacteria, showed dramatical changes at the phylum classification level after long-term drinking (**Figure 7A**), and the ratio of Firmicutes/Bacteroidetes decreased significantly, which indicate an increase in the proportion of pro-inflammatory microbe (**Figure 7B**). Moreover, the relative abundances of Allobaculum, Bacteroides, Bifidobacterium, Butyricimonas, Parabacteroides, Veillonella, Corynebacterium, Sutterella, Lactobacillus at the genus level were significantly changed after long-term drinking both in young and old rats (*P* < 0.001). And there were significant differences between the effects of alcohol on the relative abundance of old rats and that of young rats (**Figure 7C**), suggesting that the effects of alcohol on the gut microbiota of rats were age-dependent.

## Discussions

Excessive chronic alcohol consumption has various harmful effects on multiple organs, including the brain, heart, liver, muscles and the skeleton (Jongenelis et al., 2018). Numerous studies have reported that long-term drinking affects bone remodeling, leads to bone loss, and increases the risk of osteoporosis and fractures (Luo et al., 2017). Alcohol metabolism mainly occurs in the liver and is dependent on two major nicotinamide adenine dinucleotide dependent enzymes, ADH and ALDH (Muzio et al., 2012). In this study, it was found that the levels of ADH and ALDH in old rats were significantly lower than that in young rats, and alcohol intake had no significant effect on the activity of alcohol metabolizing enzymes, indicating that the ability of alcohol metabolism was only associated with age, but not with alcohol consumption. The formation of osteoporosis is that the effect of bone resorption mediated by osteoclasts is greater than the effect of bone formation mediated by osteoblasts, resulting in increased bone loss (Armas and Recker, 2012). And we found that the trabecular bone area ratio and the number of osteoblasts in rats after long-term drinking were significantly reduced, and the number of osteoclasts was significantly increased, which was consistent with existing studies (Martiniakova et al., 2018; Sarocka et al., 2018). It indicated that long-term drinking induced osteoporosis, and our research found that this bone loss was age-related.

Bones of the body are in a dynamic balance between bone formation and bone resorption, and studies have shown that TRACP-5b can be used as a marker of osteoclasts and bone resorption (Halleen et al., 2006). This study found that alcohol significantly increased the expression of TRACP-5b in serum, and this increase was more pronounced in old rats, indicating that alcohols affects bone resorption in an age-dependent manner. Bone resorption is mainly mediated by osteoclasts, and the differentiation of osteoclast depends on RANKL, a TNF family member (Fábrega et al., 2005). RANKL can bind to the homologous receptor RANK and OPG, which plays an important role in alcoholic bone metabolism (Fábrega et al., 2005), and studies have shown that RANKL can both activate mature osteoclasts and mediate osteoclast formation (Yokota, 2017). And OPG inhibits the binding and activation of RANKL and RANK, and also inhibits the development of osteoclasts and down-regulates the RANKL signaling through RANK (Grimaud et al., 2003). There were age differences in the effect of alcohol on serum OPG level, which further indicated that the effect of alcohol on bone absorption was related to age. Furthermore, the differentiation of osteoclast progenitor cells requires a series of cytokines, including NF-*к*B, c-Fos, M-CSF, and NFATc1 (Asagiri and Takayanagi, 2007). Our results showed that alcohol increased the expression of p-p65/p65, RANKL, M-CSF, c-Fos and NFATc1, indicating that alcohol promotes the differentiation of osteoclasts. In addition, the expression of these proteins is age-related. The activation degree of osteoclasts in old rats is significantly higher than that in young rats, old rats are more prone to osteoporosis than those young counterparts. Both alcohol and age are significantly associated with the activation of osteoclasts.

The link between inflammation and bone loss has been well established and both systemic and local T cell activation can lead to osteoclast production and subsequent bone loss (Kong et al., 1999). We found that T lymphocytes were activated after long-term drinking in rats, and the activation of T cell was associated with age. In addition, the overexpression of various inflammatory cytokines produced by activated T cells is one of the most important ways that leading to AOP, and proinflammatory cytokines can significantly regulate osteoclasts formation and adipocyte differentiation in the context of alcoholism and chronic alcohol exposure (Yao et al., 2001). TNF-*α*, a multifunctional inflammatory cytokine, can induce the expression of RANKL in osteoclast, or act directly on RANKL to induce osteoclast formation and promote bone resorption (Redlich et al., 2002). IL-17A regulates the autophagy activity of osteoclast precursors by activating the RANKL-JNK pathway during osteoclast formation (Ke et al., 2018). And LPS is thought to promote osteoclast differentiation and survival by producing a variety of cytokines (Hou et al., 2013). In this study, long-term drinking significantly increased the expression of TNF-*α*, IL-17A, and LPS only in old rats. Moreover, the expression of TNF-*α* and IL-17A in old rats after long-term drinking was significantly higher than that in the young rats, indicating that the effects of alcohol on the inflammatory response to promote osteoporosis was more significant in the old rats.

Gut microbiota is the normal microbial community in the human intestinal tract, which plays a variety of physiological roles such as mechanical barrier, biological barrier and immune barrier to the host (Jandhyala et al., 2015). It has been found that increased expression of TNF-*α* can promote the formation of osteoporosis, and TNF-α expression is also affected by gut microbiota (Charatcharoenwitthaya et al., 2007). Intestine and its microbiome can effectively reduce intestinal inflammation in humans by stimulating immune regulation, and help improve bone metabolic function (Mccabe et al., 2015). In this study, the changes of gut microbiota in rats with different treatment methods in different age groups were analyzed by 16S sequencing, and the results showed that the composition of gut microbiota in rats of different ages is different, and the influence of alcohol on the gut microbiota of rats of different ages is also different. Alcohol seemed to increase the value of Chao index in the old rats but reduced in the young group, although the effect is not significant, it is also an interesting phenomenon, which may be caused by the insufficient sample size, and can be further explored in the future studies. The main phylum represented the gut microbiota are Bacteroidetes, Firmicutes, Actinobacteria, Proteobacteria and Verrucomicrobia (Huttenhower et al., 2012). Among them, Firmicutes are positively correlated with calcium absorption (Whisner et al., 2016), and Bacteroides can restore the correct balance between Th1 and Th2 cells, redirects lymphoid organogenesis in germ-free animals, and directs the cellular and physical maturation of the developing immune system (Mazmanian et al., 2005). This study found that after long-term drinking, the expression of Firmicutes was significantly decreased, and the expression of Bacteroides was significantly increased, and the proportion of Firmicutes/Bacteroides was also significantly decreased, indicating an increase in the proportion of pro-inflammatory microbe accompanied by long-term drinking. In addition, long-term drinking was associated with significant changes in the relative abundance of many gut microbiota, and the effects of alcohol on gut microbiota in rats were age-related.

## Conclusion

In conclusion, this study found that long-term alcohol consumption could affect the expression of bone metabolism regulating hormones, induce the activation of osteoclast differentiation and influence the composition of gut microbiota in rats. And alcohol triggered the activation of T cells in the bone marrow directly or indirectly by regulating the changes of gut microbiota to produce cytokines, and further activate the osteoclasts. In addition, osteoporosis in the old rats was more serious than that of the young rats, which may be related to the composition of the gut microbiota. The gut microbiota of old rats has a lower ability to regulate immunity and hormones, while the gut microbiota of young rats has a higher diversity and its regulatory ability after drinking is stronger than that of the old rats.

## Data Availability Statement

The raw data supporting the conclusions of this article will be made available by the authors, without undue reservation.

## Ethics Statement

The animal study was reviewed and approved by Sichuan Province Experimental Animal Management Committee.

## Author Contributions

ZH conceived and designed the research. MC and BT performed most of the experiments and wrote the paper. XW and FL performed parts of the experiments. XW, FL, and FW analyzed the data. All authors contributed to the article and approved the submitted version.

## Funding

This work was supported by the first batch of key R&D projects of Sichuan Provincial Science and Technology Department in 2020 (2020YFS0416).

## Conflict of Interest

The authors declare that the research was conducted in the absence of any commercial or financial relationships that could be construed as a potential conflict of interest.
